# Unravelling the thermal behavior and kinetics of unsaturated polyester resin supplemented with organo-nanoclay†

**DOI:** 10.1039/d3ra06076d

**Published:** 2024-01-02

**Authors:** Ayoub Chencheni, Samir Belkhiri, Ahmed Fouzi Tarchoun, Amir Abdelaziz, Wissam Bessa, Youcef Boucheffa, Djalal Trache

**Affiliations:** a Energetic Propulsion Laboratory, Teaching and Research Unit of Energetic Processes, Ecole Militaire Polytechnique BP 17, Bordj El-Bahri Algiers 16046 Algeria tarchounfouzi@gmail.com; b Energetic Materials Laboratory, Teaching and Research Unit of Energetic Processes, Ecole Militaire Polytechnique BP 17, Bordj El-Bahri Algiers 16046 Algeria djalaltrache@gmail.com; c Faculty of Chemistry, University of Sciences and Technology Houari Boumediene Bab El-Zouar Algiers Algeria

## Abstract

The integration of nanoclays within polymeric systems to develop high-performance materials is an emerging research field that has garnered significant attention. In this context, an organically modified montmorillonite (OMMT) is utilized as a reinforcing agent for unsaturated polyester resin (UPR) with loads of 1%, 3%, and 5 wt%. The modification of montmorillonite nanoclay (MMT) using a quaternary ammonium compound is performed through an effective repetitive modification process under reflux conditions. The curing behavior of the unsaturated polyester resin containing organically modified clay catalyzed with methyl ethyl ketone peroxide (MEKP) initiator and promoted by cobalt naphthenate accelerator is investigated using dynamic differential scanning calorimetry (DSC) followed by kinetic analysis using isoconversional methods. The dynamic DSC curing curves showed a bimodal exothermic peak, where two independent reactions, namely, redox and thermal decomposition of the initiator occurred. In this study, novel insights into the curing reaction of the studied UPR and UPR/OMMT systems have been revealed through the application of the Trache-Abdelaziz-Siwani (TAS) and Sbirrazzuoli (VYA/CE) isoconversional methods. These methods have enabled the elucidation of the intricate mechanisms and phenomena that impact the curing reaction, including the dilution effect in the redox reaction and the diffusion phenomenon at the end of the thermal decomposition reaction. The incorporation of nanoclay into unsaturated polyester resin (UPR) resulted in a reduction in the activation energy for both the redox and thermal reactions. Specifically, the energetic barrier decreased from 93.85 and 101.58 kJ mol^−1^ for pristine UPR to 60.71 and 72.93 kJ mol^−1^ for UPR/OMMT-5 in the redox and thermal reactions, respectively. The addition of OMMT caused a significant decrease in the pre-exponential factor. The values of UPR/OMMT-5 were 2.75 × 10^5^ and 5.50 × 10^6^ for the redox and thermal decomposition reactions, respectively, compared to 1.41 × 10^12^ and 5.13 × 10^13^ for UPR. The thermogravimetric analysis demonstrated that UPR/OMMT systems were more stable than UPR.

## Abstract 2

Montmorillonite nanoclay (MMT) was modified using a quaternary ammonium compound. The incorporation of nanoclay into unsaturated polyester resin (UPR) was shown to be more stable and had reduction in both the energetic barrier and pre-exponential factor for redox and thermal reactions.

## Graphical abstract

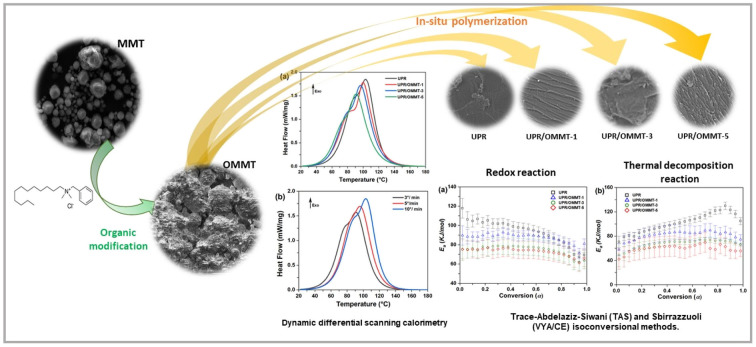

## 1. Introduction

Nowadays, nanoclay-based polymeric systems have gained considerable attention from the research community owing to their outstanding properties such as thermal stability,^1^ mechanical strength,^2^ and barrier properties.^3^ Indeed, the nanoscale dispersion of the filler phase in the polymer matrix leads to tremendous interfacial contact of the nanoparticles with the polymer matrix, resulting in synergistic improvements in the composite features.^4^ Additionally, surface modification of nanoclays with organic molecules has proven to be an effective technique for enhancing the interaction with organic matrices.^5^ Smectite, particularly montmorillonite (MMT), is a commonly used nanofiller due to its exceptional properties such as high cation exchange and adsorption capacity as well as swelling behavior.^6–8^ The crystal structure of MMT consists of octahedral alumina and tetrahedral silica sheets, where the central atoms (Al^3+^ and Si^4+^) are substituted with lower valence ions (Mg^2+^ and Fe^2+^).^9^ Organic modification of MMT is typically achieved through ion–exchange reactions, including quaternary alkylammonium^10,11^ or alkylphosphonium^12,13^ and anionic surfactants such as carboxylic acid salts.^14,15^

Unsaturated polyester resins (UPRs) emerge as a cost-effective and chemically resistant substitute for other resins. Their market share is expected to grow significantly, and this trend is expected to continue.^16^ UPRs have diverse applications in multiple sectors, encompassing transportation, electrical appliances, construction, and buildings.^17^ The incorporation of inorganic fillers such as nanoclays has proven, in multiple studies, to yield significant enhancements in mechanical strength, thermal stability, and barrier properties.^18–20^ Nevertheless, the curing behavior of such systems using thermo-kinetic approaches is not fully explored and further research activities are required. One of the most widely used techniques for studying the kinetics of the cure reaction of thermosetting resins is differential scanning calorimetry (DSC) in dynamic mode followed by kinetic analysis using isoconversional methods. In this context, Poorabdollah *et al.*^21^ investigated the curing behavior of an unsaturated polyester resin containing organically modified clay catalyzed with methyl ethyl ketone peroxide (MEKP) initiator and promoted by cobalt naphthenate accelerator. The dynamic DSC curing curves showed a bimodal exothermic peak, where two independent reactions, namely, redox and thermal copolymerizations were assumed. The kinetic parameters were calculated by using the autocatalytic model using the Downhill simplex method and the Runge–Kutta algorithm for each reaction. The results showed that the addition of nanoclay decreased the activation energy and pre-exponential factor of the redox reaction compared to that of the neat UP resin. The pre-exponential factor of the first reaction for UP/OMMT was less than that of the neat UP. In another study, the same authors^22^ presented a method for optimizing the cure cycle of unsaturated polyester resin and polyester resin containing 3% wt. nanoclay Cloisite® 10 A (UP/10 A) and nanoclay Cloisite® 30B (UP/30B) in thin components. The study considered four different kinetic models, including the advanced iso-conversional method, Kamal method, Kamal method with Chern diffusion factor, and Kamal method with Sbirrazzuoli diffusion factor. The results showed that the addition of nanoclay decreased the cure cycle duration as well as the maximum curing temperature. The co-catalytic effect of the organomodified nanoclay on the curing of the unsaturated polyester resin is evidenced by other studies using isoconvensional and other kinetic models.^23–25^

The advanced isoconversional method has been proposed as a reliable kinetic method for the treatment of thermoanalytical data. It has been shown to be effective in elucidating complex reaction mechanisms and identifying rate-limiting steps,^26^ however, obtaining kinetic parameters with real physical meaning in the case of complex reactions is not straightforward^27^ and the use of simple and easy-to-use method as Kamal method is not suitable for complex reactions. On the other hand, the advanced isoconversional method requires a large amount of experimental data. The need to use more kinetic models arises from the fact that different models can provide different kinetic parameters and optimal cure cycles.^26^ This approach can provide a more comprehensive understanding of the curing behavior of nanoclay-reinforced unsaturated polyester resin. Therefore, the current study aims to investigate the curing behavior of unsaturated polyester resin doped with 1%, 3%, and 5 wt% of MMT nanoclay that has been modified using a quaternary ammonium compound through a repetitive modification process under reflux conditions. DSC experiments were undertaken to determine the thermal behavior of the studied samples under different heating rates and with a fixed initiator/promoter ratio. In addition, their kinetic parameters were determined using isoconversional kinetic methods, namely, Trache-Abdelaziz-Siwani (TAS) and Sbirrazzuoli methodology (VYA/CE), and by assuming the occurrence of two independent reactions, *i.e.*, redox and thermal decomposition. This study will provide further insight into the elucidation of complex cure mechanisms of UPRs systems.

## 2. Experimental

### 2.1. Materials

Montmorillonite (MMT) and benzododecinium chloride salts (DDBAC) are purchased from Sigma Aldrich, USA. The unsaturated isophthalic polyester resin (Norsodine) with styrene content of up to 35 vol% is provided by MPC PROKIM Industries, Tunisia. The initiator methyl-ethyl ketone peroxide in dimethyl phthalate (Butanox M-50) is purchased from Akzo Nobel, Netherlands, while the accelerator cobalt naphthenate is acquired from BOYTEK, Turkey.

### 2.2. Material synthesis

The organic modification process is a technique used to modify the properties of clay minerals, such as Montmorillonite (MMT), by intercalating organic molecules into the clay structure. The process is typically carried out under reflux. The process starts by mixing 5 grams of MMT with a quantity of DDBAC equivalent to twice the MMT's cation exchange capacity (CEC) in 1000 milliliters of deionized water. Next, 1 milliliter of 37% hydrochloric acid (HCl) is added to the solution to dissolve and ionize the organic surfactant. The mixture is then heated at 80 °C under continuous magnetic stirring for 3 hours. This step is essential to facilitate the intercalation of the organic molecules into the clay structure. After the intercalation process is completed, the solution is filtered to separate the modified clay (OMMT) from the excess DDBAC. The OMMT is then rinsed several times with deionized water at 40 °C to remove any residual DDBAC. The modification process is repeated several times. In the end, the recovered solid is dried overnight in an oven at 80 °C, and the modified clay is crushed and stored in a desiccator.

The desiccated organoclay is incorporated into the UPR with a ratio of 1, 3 and 5% by mass of the isophthalic unsaturated polyester resin. Before this step, the UPR is promoted by mixing the pristine resin with cobalt octoate promoters at a ratio of 0.2% wt. while stirring at 1500 rpm at 50 °C for 1 hour. The mixture is then cooled to room temperature before the hardener, MEKP, is added at a ratio of 2% wt. of the resin under stirring. The samples are placed in a PTFE mold and cured in an oven at 45 °C for 24 hours to improve the crosslinking of the resins and prevent the formation of air bubbles. The final step involves a post-curing at 90 °C for 6 hours to complete the crosslinking process of the UPR/OMMT systems.

### 2.3. Characterization

X-ray diffraction (XRD) measurements of MMT, organoclays, and UPR/OMMT systems are conducted on a PANalytical X'Pert Pro diffractometer with Cu K-alpha1 radiation (*λ* = 1.540598 Å), operating at 45 kV and 40 mA. The Fourier Transform Infrared (FTIR) analyses are conducted with a Thermo Nicolet Nexus 4700 FT-IR Spectrometer within the range of 4000–400 cm^−1^ at a resolution of 4 cm^−1^. Raman spectra were collected on a Raman spectrometer-type Thermo Fisher DXR2. Differential scanning calorimetry (DSC) measurements are performed using a PerkinElmer 8000 DSC instrument with 20 ml min^−1^ nitrogen flow and at heating rates of 3, 5 and 10 K min^−1^. The uncured samples are carefully placed in an aluminum capsule after adding the hardener. Thermogravimetric Analyses (TGA) are carried out on Q500 TA instruments with a nitrogen flow of 10 ml min^−1^ and a heating rate of 10 °C min^−1^ from room temperature up to 700 °C. The morphology of the studied materials is investigated by Scanning Electron Microscopy (SEM) measurements using FEI Quanta 600 device, equipped with EDAX Ametek X-ray detector.

### 2.4. Theoretical section

According to the International Committee of Thermal Analysis and Calorimetry (ICTAC), the thermal process is typically described by the following kinetic equations, where the temperature dependence of the rate constant *k*(*T*) is expressed by the Arrhenius equation.^28^1
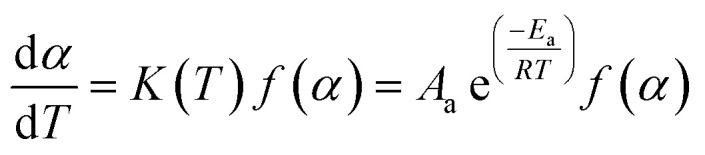
2
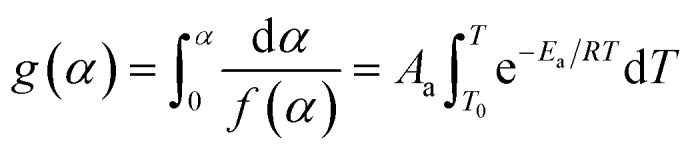


Therefore, *A*_a_, *E*_a_, *f*(*α*) and *g*(*α*) are the preexponential factor, activation energy, differential and integral forms of the model, respectively, while *α* presents the extent of conversion (0 < *α* < 1). The collection of these parameters is termed the kinetics triplet (*E*_a_, Log(*A*), *g*(*α*)).

The value of *α* can be determined from the temperature integral of the DSC thermograms, obtained at various heating rates, by using (eqn (3)), in which Δ*H* is measured at change and Δ*H*_total_ is the total reaction heat.^29,30^3
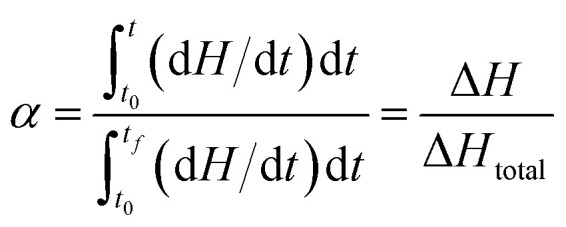


Calculation of the kinetic triplet was accomplished using isoconversional models, namely, Trache-Abdelaziz-Siwani (TAS),^31^ and Vyazovkin's method (VYA) coupled with the compensation effect approach (CE).^32^

## 3. Result and discussion

### 3.1. Characterization of organically modified montmorillonite

**Fig. 1:**
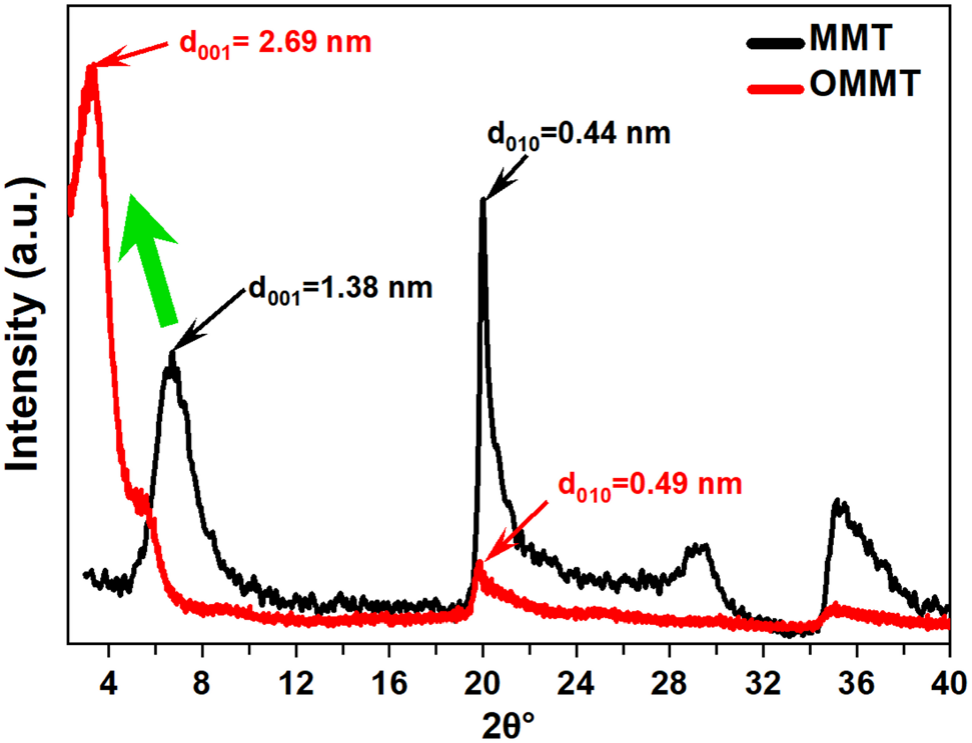
X-ray diffraction patterns of MMT and OMMT nanoclays.

XRD analysis is used to investigate the crystalline structure of unmodified and modified MMT samples. The most commonly observed crystallographic planes in montmorillonite are the basal (001) and the edge (010) planes. The basal plane is the flat surface formed by the repeating silicate sheets, while the edge plane is perpendicular to the basal plane and runs along the edges of the mineral grains.^26^ As shown in Fig. 1, A significant shift in the MMT peak (2*θ* = 6.39°) toward lower angles is observed for OMMT (2*θ* = 3.27°). This shift in the peak position is attributed to the intercalation of organic compounds into the montmorillonite structure. The intercalation of these compounds leads to an increase in the interlayer spacing between the silicate sheets in the mineral. This expansion is supported by the elevated basal distance *d*_001_ values of the modified montmorillonite sample, which are determined to be 2.69 nanometers. Additionally, Noteworthy modifications in the intensity and shape of the edge plan peaks (010), along with a slight shift in position, suggest changes in the crystal structure, which can be attributed to the substitution of the structural OH groups at the edges with anionic surfactants.^33^

**Fig. 2:**
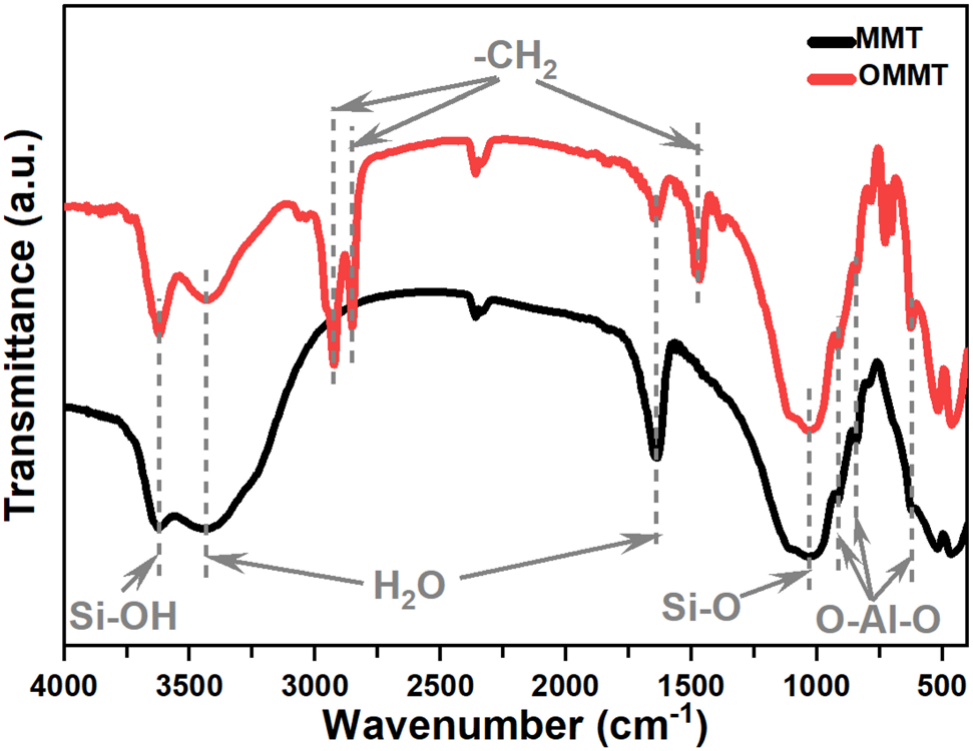
FTIR spectra of MMT and OMMT nanoclay.

The Fourier Transform Infrared spectra of MMT and OMMT are presented in the spectral range of 4000–400 cm^−1^ in Fig. 2. The symmetric stretching vibration of the hydroxyl groups observed at 3407 cm^−1^, and the bending-in-plane vibration of the H–O–H at 1634 cm^−1^ correspond to the surfactants replacing H_2_O in the clay interlayer spaces.^28^ The vibrations of the interlamellar OH groups in MMT shifted from 3625 cm^−1^ to 3628 cm^−1^ due to the removal of structural hydroxyl groups from the Si–OH and Al–OH sites.^29^ The Si–O stretching large band noticed at 1033 cm^−1^ is affected by the surfactant intercalated into the interlayer space of montmorillonites.^34^ The bands at 915, 793, and 521 cm^−1^ that corresponded to the aluminosilicate octahedral layers are also affected by the modification in terms of position and intensities.^35^ Furthermore, the FTIR spectrum OMMT reveals the presence of two strong bands near 2849–2935 cm^−1^, which correspond to asymmetric (*ν*_as_(CH_2_)) and symmetric (*ν*_s_(CH_2_)) stretching modes.^36^ These frequencies are well-known to be sensitive to the conformational changes in the hydrocarbon chains,^34,37^ in addition, the peaks obtained at 1469 cm^−1^ are assigned to the scissoring vibrations of –CH_2_ as a result of intermolecular attractions between adjacent alkyl chains in the interlayer space.^38^ These results highlight the presence of the organic modifier within MMT galleries.

**Fig. 3:**
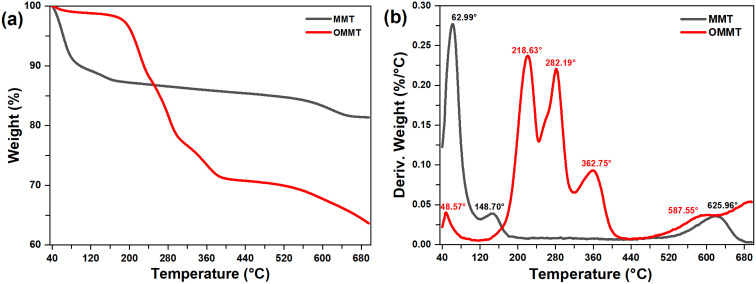
(a) TGA and (b) DTG thermograms of MMT and OMMT nanoclay.

The thermal stability and decomposition temperatures of the intercalated clay minerals are determined by TGA analysis. The TGA and DTG thermograms of MMT, MMT-SOD, and MMT-DDBAC are shown in Fig. 3. During the first dehydration observed at 63.0 °C, 10.7% of the MMT weight is lost through the evaporation of water molecules adsorbed in pores. This mass loss is followed by a second dehydration noticed at 148.7 °C, which is due to the departure of the interlayer water molecules.^39^ Nevertheless, the same behavior is also observed for OMMT at lower temperatures 48.6 °C, with lower mass losses by 1.1%. The end of the OMMT's second-stage decomposition is characterized by 11.26% mass loss caused by the thermal decomposition of the alkyl tails (–CH_2_).^40^ In the third step, the thermal degradation of OMMT slowed down slightly until 320 °C by 10.5% mass, due to the decomposition of the remaining alkyl chains and carboxylates.^40^ The fourth and fifth steps, involving mass loss noticed between 320 and 700 °C, are due to the dihydroxylation of OH groups in the octahedral and tetrahedral layers and the degradation of organic chains bonded to these OH groups.^41^

As shown in Table 1, a substantial reduction of the inorganic residue is constated from 81.38% for MMT to 63.63% for OMMT. These significant reductions highlight the permanent structure modification of the MMT after intercalation with DDBAC.

**Table 1:** TGA results for MMT and OMMT nanoclay

Sample	1st step		2nd step		3rd step		4th step		5th step		Residue (%)
	*T*_D_ (°C)	*W*_*t*.los_ (%)	*T*_D_ (°C)	*W*_*t*.los_ (%)	*T*_D_ (°C)	*W*_*t*.los_ (%)	*T*_D_ (°C)	*W*_*t*.los_ (%)	*T*_D_ (°C)	*W*_*t*.los_ (%)	
MMT	62.99	10.64	148.70	02.03	—	—	—	—	625.96	5.94	62.99
OMMT	48.57	01.15	218.63	11.26	282.19	10.80	362.75	5.90	587.55	7.14	48.57

**Fig. 4:**
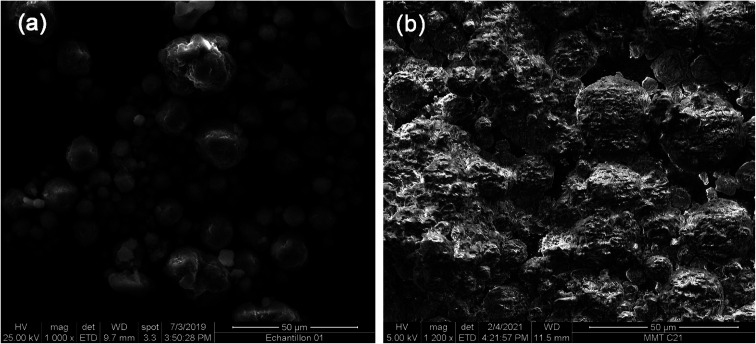
SEM images of (a) MMT and (b) OMMT nanoclay.

The SEM Micrographs of MMT and OMMT are presented in Fig. 4a and b. It can be seen that MMT has a typical lamellar structure, consisting of thin and flat platelets with well-defined edges and grain boundaries.^35^ These platelets have a high surface area and a strong affinity for water molecules, which enables them to swell and absorb large amounts of liquids. However, after organic modification, the lamellar structure of MMT is disrupted and the platelets tend to aggregate into larger particles with irregular shapes and sizes. This indicates that the organic compounds have penetrated the interlayer spaces of MMT and acted as a binder, preventing the platelets from separating and reducing their water absorption capacity.

### 3.2. Characterization of elaborated UPR/OMMT systems

**Fig. 5:**
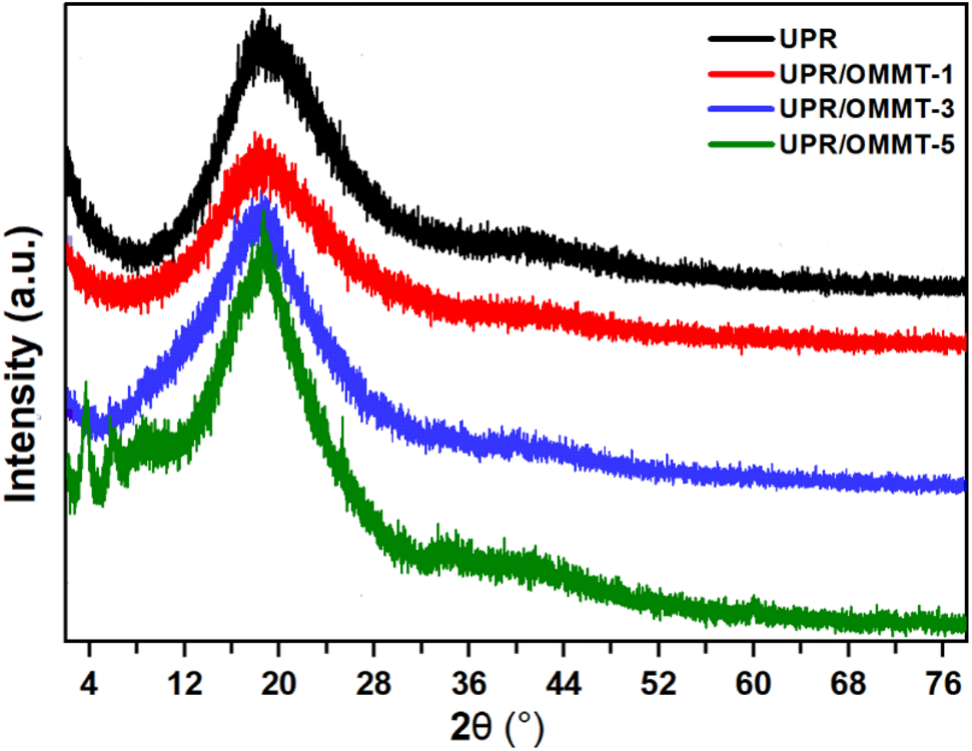
X-ray diffraction patterns of pristine UPR and UPR/OMMT systems with loadings of 1, 3, and 5%.

The X-ray diffraction (XRD) patterns of pristine UPR and UPR/OMMT systems are shown in Fig. 5. The UPR pattern displays a broad peak around 2*θ* = 20°, indicating the partial crystallinity and high amorphousness of the resin. This peak shape differs from the diffuse one of an amorphous sample and the sharp one of a crystal.^42^ The patterns of modified UPR with a load of 1% and 3% of OMMT exhibit a slight shift in peak position to a lower scattering angle, suggesting the intercalation of nanoparticles into the matrix. These results imply that the OMMT is well dispersed within the resin matrix, enhancing the interfacial adhesion between the filler and the matrix.^37^ Moreover, a slight decrease in peak width and an increase in peak intensity are observed for both UPR/MMT and UPR/OMMT systems, implying an increase in the number of crystalline domains and a decrease in the crystallite size of UPR in the nanocomposite.^43^ Two supplementary broad peaks are apparent in the UPR-based nanocomposite XRD pattern loaded with 5% OMMT, which is due to the relatively high nano-charge load in the UPR matrix. The dispersion protocol used in this study represents a limitation threshold for obtaining a homogenous dispersion of OMMT in the matrix.

**Fig. 6:**
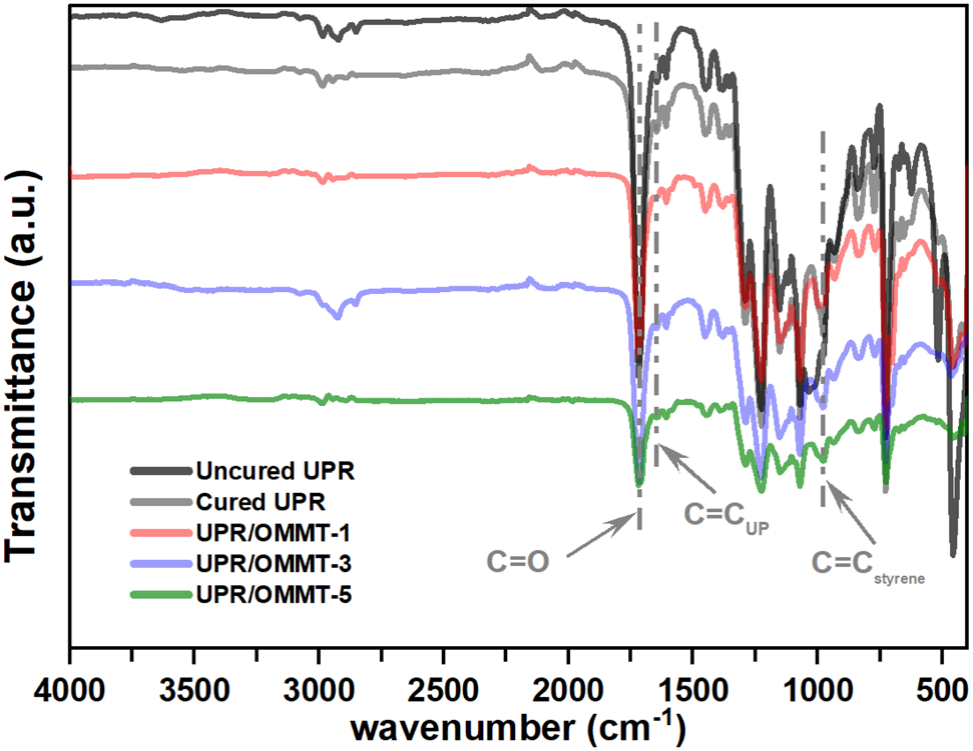
FTIR spectrum of uncured and cured UPR, UPR/OMMT systems with loadings of 1, 3, and 5%.

In Fig. 6, the FTIR spectra of uncured and cured unmodified UPR are compared to modified unsaturated polyester resin with a load of 1%, 3%, and 5% of OMMT. The FTIR spectra of pristine UPR reveal the presence of characteristic features, including aromatic rings at 3081, 3077, 1600, and 1579 cm^−1^, and a pass band at 1071 cm^−1^.^41^ The transmission band at 1725 cm^−1^ corresponds to the C=O stretching vibration of ester groups.^44^ Additionally, allyl groups are observed at 1648, 979, and 744 cm^−1^. Methyl groups are apparent at 2957 and 1451 cm^−1^, while methylene groups are observed at 2883 and 775 cm^−1^. Furthermore, the FTIR spectra indicate the presence of the aromatic ring of styrene at 1489 and 669 cm^−1^.^41^ The vinyl C=C band at 1648 cm^−1^, which comes from the double bond in styrene, becomes less intense in cured UPR samples. When OMMT is present, the band disappears entirely. The vinyl C–H band at 1410 cm^−1^ from styrene also decreases and is barely visible in the post-cured sample.^45^ The saturated C–H band at 1460 cm^−1^ increases slightly in intensity because the vinyl groups turn into saturated C–H groups during cross-linking. The aromatic C=C band at 1600 cm^−1^ also decreases in intensity, but not as much as the C=C band. This is partly due to styrene evaporating from the open mold at the start of crosslinking and changes in the spectral environment of the styrene molecule caused by the nanoparticle during curing.^45^

**Fig. 7:**
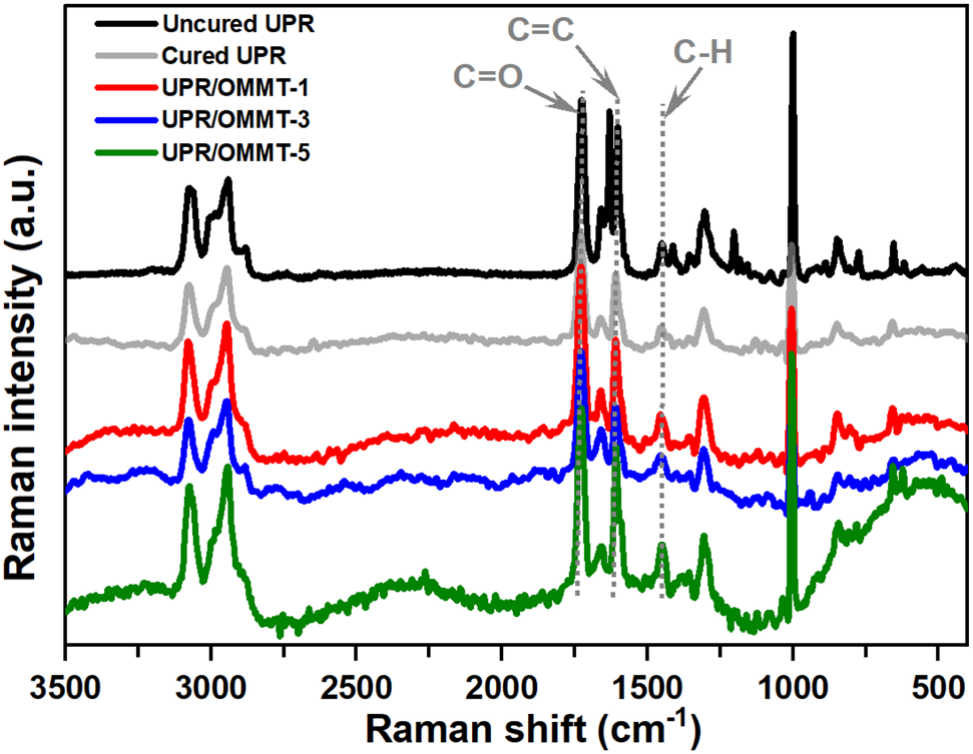
Raman spectrum of uncured and cured UPR, UPR/OMMT systems with loadings of 1, 3, and 5%.

Raman spectroscopy is employed to investigate the spectral changes in the 400–3500 cm^−1^ region for both uncured and cured samples. The obtained results are presented in Fig. 7. The observed increase in the spectral slope is attributed to the fluorescence of samples, necessitating the use of separate baselines for the functional groups to calculate their intensity accurately. The intensity of the vinyl C=C band at 1630 cm^−1^, originating from the double bond in styrene, disappeared entirely after the post-curing process. Additionally, the intensity of the vinyl C–H band at 1410 cm^−1^, which also originates from styrene,^46^ reduced significantly and became challenging to observe in the post-cured sample. In contrast, the intensity of the carbonyl band at 1730 cm^−1^ remained constant and served as a reliable internal standard. Furthermore, the intensity of the saturated C–H band at 1460 cm^−1^ increased slightly due to the transformation of vinyl C–H groups into saturated C–H groups during the crosslinking reaction.^46^ Interestingly, the intensity of the aromatic C=C aromatic band at 1600 cm^−1^ also decreased, even though not as significantly as the C=C_UP_ band.

**Fig. 8:**
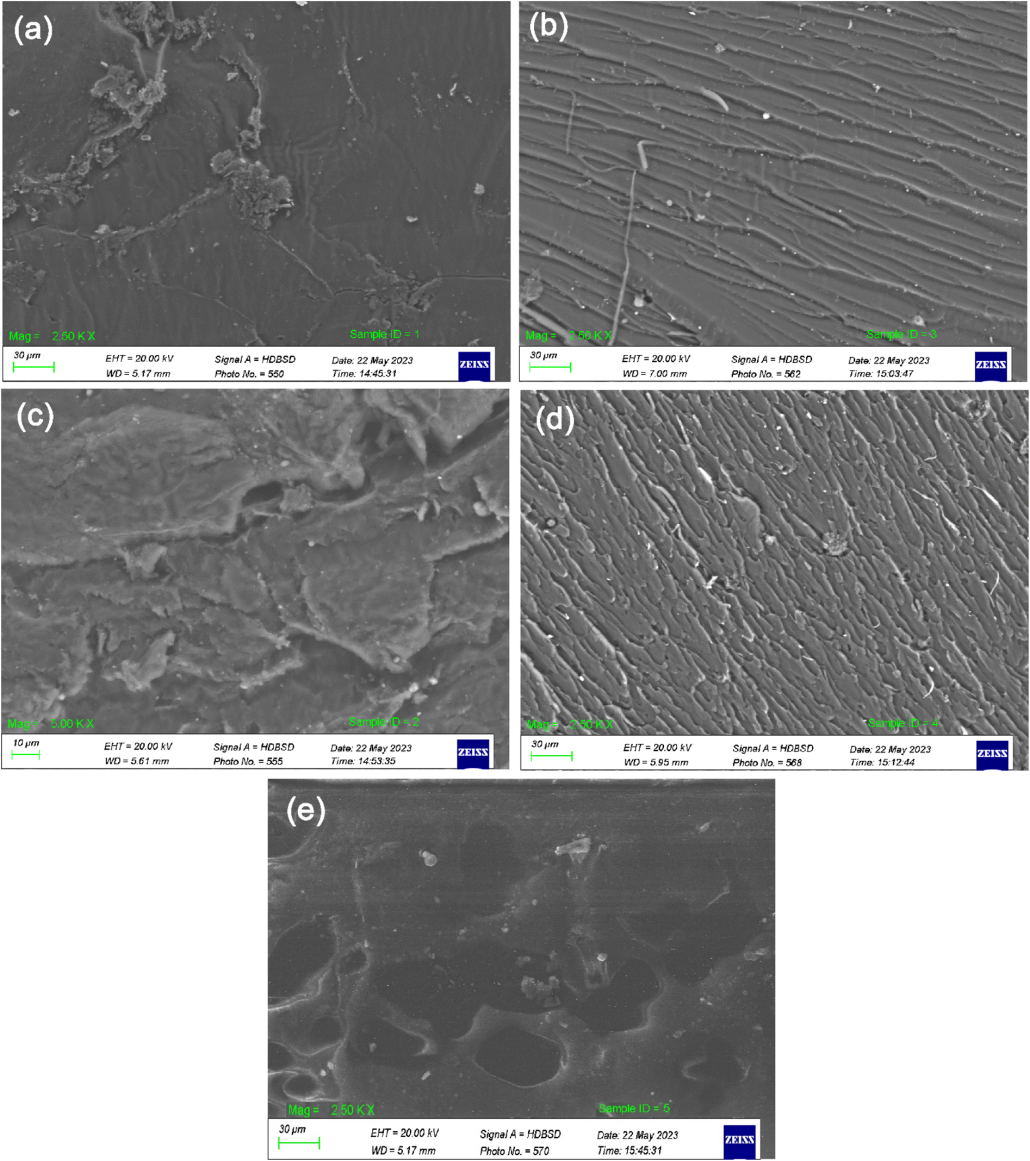
SEM images of (a) UPR and UPR/OMMT with a load of (b) 1%, (c) 3% (d) 5% and (e) UPR/MMT 1%.

The SEM images of the unmodified UPR, UPR/MMT, and UPR/OMMT systems are shown in Fig. 8a–e. The fracture surfaces of pure polyester and polyester-nanoclay systems show significant differences. The surface of pure polyester is completely smooth and lacks any features, which is characteristic of glassy material. This suggests that cracks propagate quickly and the material has low fracture toughness, as shown in Fig. 8a. According to the SEM images Fig. 8b–d, the morphology of the fractured surfaces becomes coarser as the load of nanoclay increases. The generation of microcracks in the presence of nanoclay platelets is due to the presence of stress concentrations in the composite material. The nanoclay platelets act as effective barriers that impede the progression of cracks throughout the composite material. The formation of numerous microcracks creates a tortuous path for crack propagation, which effectively enhances the material's fracture toughness. The overall fracture surface area expands due to the presence of these microcracks. This phenomenon has been observed in polyester-nanoclay nanocomposites, where the addition of nanoclay platelets significantly influences the morphology of the fractured surfaces.^47,48^

### 3.3. Non-isothermal curing reaction of unsaturated polyester systems

**Fig. 9:**
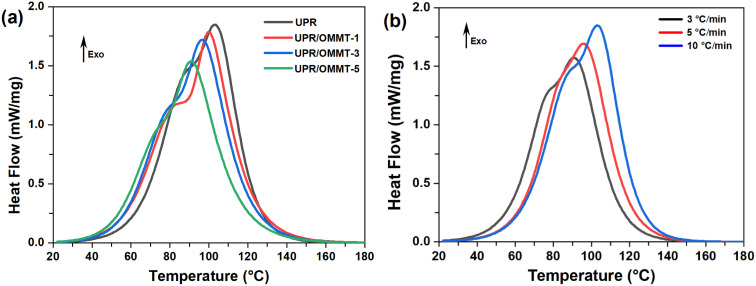
DSC thermograms of (a) UPR and UPR/OMMT systems at 10 °C min^−1^, (b) pristine UPR at 3, 5 and 10 °C min^−1^.

The non-isothermal differential scanning (DSC) thermograms of UPR and UPR/OMMT systems with 1, 3, and 5% loads, heated at a rate of 3, 5 and 10 °C min^−1^, are depicted in Fig. 9a and b. The exothermic peaks exhibit a bimodal shape curve, which results from the overlapping of two individual peaks that can be attributed to two separate reactions. It is worth noting that cobalt naphthenate promotes the polymerization reaction of unsaturated polyester resin systems, causing the reaction to be performed at low temperatures. Therefore, it is assumed that the first reaction that occurred at a lower temperature is due to the redox decomposition of the initiator, whereas the second one that appeared at a higher temperature is related to its thermal decomposition. The DSC curves reveal that the presence of nanoclay has a discernible effect on the curing process, as evidenced by the differences in the DSC curves of the neat resin and UPR/OMMT systems.^49^ Quantitative data on the curing thermal behavior of the pristine and loaded UPR, curing heat release (Δ*H*_T_), and peak cure temperatures (*T*_p1_) and (*T*_p2_) are reported in Table 2. This latter shows that the change in Δ*H*_T_ remains relatively constant with increasing heating rate, falling within the range of 265–395 J g^−1^, which is consistent with previous research on polymerization heat for which the values ranged from 290 to 426 J g^−1^ for pure UPR and its nanocomposites.^50,51^ The differences in the values may be attributed to the different types of UPR and the free radical initiator systems used in each study. In the UPR/OMMT systems, a slight reduction of Δ*H*_T_ is observed compared to the neat resin. This decrease in the heat of the reaction can be attributed to the steric or dilution effect caused by the dispersed OMMT, which may reduce the extent of the cross-linking reaction.^52^ The peak temperatures (*T*_p1_ and *T*_p2_) of curing for all samples increase with the heating rate, as is commonly observed for dynamic methods. However, the peak temperatures decrease with the content of nanoclay, indicating a slight increase in the reaction kinetics.^53–55^

**Table 2:** Overall reaction heat (Δ*H*_T_), peak cure temperatures (*T*_p1_ and *T*_p2_) for non-isothermal UPR and UPR/OMMT curing reaction

Samples	Heating rate (*β*) (°C min^−1^)	*T*_p1_ (°C)	*T*_p2_ (°C)	Δ*H*_T_ (J g^−1^)
UPR	3	78	90	375
	5	86	96	383
	10	89	103	395
UPR/OMMT-1	3	76	86	350
	5	81	93	366
	10	84	99	370
UPR/OMMT-3	3	70	84	292
	5	77	87	320
	10	79	96	345
UPR/OMMT-5	3	66	82	265
	5	84	95	270
	10	89	102	288

In order to acquire a better insight into the impact of different OMMT loads on the curing process of the UPR polymer, a deep investigation of the curing kinetics has been conducted using isoconversional methodology, where the kinetic parameters that characterize their curing process *i.e.* the activation energy (*E*_a_), the pre-exponential factor (Log(*A*)), and the mechanisms of thermal decomposition *g*(*α*) have been determined. The variation of *E*_a_ and Log(*A*) of pristine UPR and UPR/OMMT systems has been calculated based on TAS and VYA/CE methods. The details of the computing approach and calculation procedure can be found in our previous works.^56,57^ The calculations are performed by MATLAB software within a conversion range of 0.02 to 0.98 with a step of 0.02 and according to the recommendation of the International Confederation for Thermal Analysis and Calorimetry (ICTAC).^48^

**Fig. 10:**
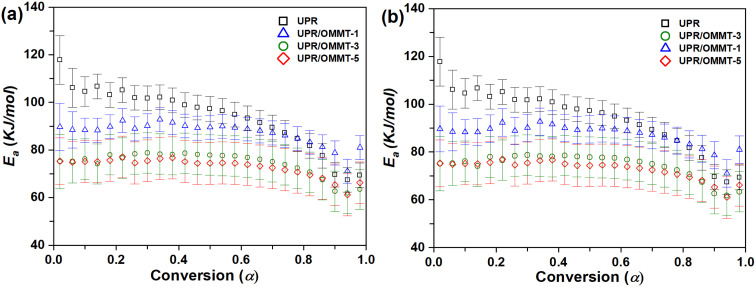
Activation energy *versus* conversion for the redox decomposition of peroxide (first reaction) in UPR and UPR/OMMT systems using (a) TAS and (b) VYA/CE method.

In this study, the calculation of the kinetic parameters is performed by assuming the presence of two separate reactions for pristine UPR and UPR/OMMT systems. The first reaction corresponds to the redox decomposition of the initiator, while the second one is assigned to its decomposition. Fig. 10a and b illustrate the dependence of *E*_a_ on the conversion degree using TAS and VYA/CE methods, respectively, at various heating rates for the first reaction related to the redox decomposition reaction of the peroxide (initiator) by cobalt naphthenate.

The reactions involved in the redox decomposition of the peroxide by cobalt naphthenate are as follows:^58^4
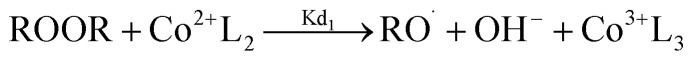
5
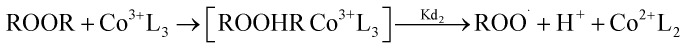
where ROOR, Co^2+^(Co^3+^), ROO˙ and L denote the initiator, promoter, free radicals, and naphthenate or octoate ligand, respectively.

The curing mechanism of neat UPR and nanoclay-filled UPR systems (UP/OMMT-1, UPR/OMMT-3, and UPR/OMMT-5) is complex, as shown in Fig. 10. The variation trend of the activation energy with conversion degree is different for these systems. Neat UPR has high initial activation energy values that decrease continuously with conversion degree to reach a minimum at *α* ≈ 0.94. On the other hand, UPR/OMMT systems show an initial progressive increase in the activation energy within the range of *α* ≈ 0.02–0.40, which decreases quickly within the conversion range of 0.4–0.5 due to the dilution effect caused by dispersed OMMT.^59^ However, for higher values of *α* (until *α* ≈ 0.94) and higher temperature values, OMMT seems to catalyze the curing reaction and partially conceal its dilution effect. This behavior is observed even for *α* values where the phenomena of gelling, vitrification, and high viscosity in the reaction medium begin to take place, hence controlling the reaction process.^60^

**Fig. 11:**
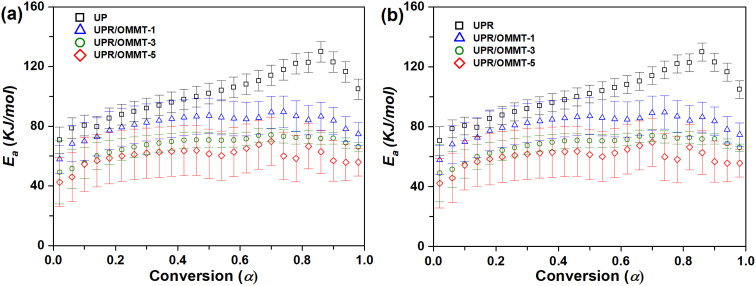
Activation energy *versus* conversion for the thermal decomposition of peroxide (second reaction) in UPR and UPR/OMMT systems using (a) TAS and (b) VYA/CE method.

For the second reaction, which represents the thermal decomposition of the initiator,^25,61^ the variation trend of the activation energy with conversion degree for UPR and UPR/OMMT systems was investigated. The results using TAS and VYA/CE methods are presented in Fig. 11a and b, respectively. The activation energy curve displays two distinct regions based on the degree of conversion. The first region, within the range of *α* ≈ 0.02–0.80, corresponds to the initial dominance of peroxide thermal decomposition over redox reaction.^25,61^ In this region, there is a rapid increase in the activation energy, with a maximum value of approximately 80 to 90 kJ mol^−1^ for UPR/OMMT systems and 90 to 110 kJ mol^−1^ for pristine UPR, which is proposed for the thermal decomposition of the MEKP peroxide.^61^ The increase in activation energy of the UPR systems is attributed to the intensive development of a three-dimensional cross-linked network and an increase in molecular motion restrictions. These factors make the collision and diffusion of molecules more difficult, requiring higher energy for polymerization.^62^

The second region, characterized by conversion degrees greater than 0.8, is associated with the dominance of the diffusion phenomenon over the thermal decomposition reaction.^24,25^ As a result, the activation energy for pristine UPR and all three UPR-OMMT systems experiences a sudden decrease. In this final stage, the movement of the reacting components is limited due to the increasing density of the resin network. Consequently, these components need to penetrate into the resin network in order to collide with each other. Thus, the diffusion phenomenon becomes highly significant towards the end of the reaction. The diffusion process in unsaturated polyester resin is complex, and influenced by various factors affecting the curing reaction.^24^ The participation of peroxide, cobalt naphthenate, alkyd chains, and styrene molecules in the curing reaction of unsaturated polyester resin simultaneously further complicates the diffusion phenomenon. Any changes in the properties of these components or the addition of an external agent can alter the diffusion behavior during the curing process, particularly in the region where diffusion predominates.^58^

The pre-exponential factor values for the redox and thermal regions (A_1_ and A_2_) for UPR and UPR/OMMT systems calculated by TAS and VYA/CE methods are given in Table 3 and the ESI.† By comparing the pre-exponential factor values for the redox and thermal reactions of UPR and UPR/OMMT systems, it can be inferred that at higher temperatures, the movement of the organic peroxide, polyester resin, and styrene molecules is faster.^61^ Therefore, the pre-exponential factor of the thermal reaction, which indicates the number of collisions of reactionary components, is higher than the pre-exponential factor of the redox reaction.

**Table 3:** Kinetic parameters obtained by TAS and VYA isoconversional methods for pristine UPR and UP/OMMT systems containing 1, 3, and 5 wt%

Sample	Method	*E*_a1_ (kJ mol^−1^)	*E*_a2_ (kJ mol^−1^)	A_1_ (S^−1^)	A_2_ (S^−1^)	Integral reaction mechanism (*g*(*α*))
UPR	TAS	93.85	101.58	1.41 × 10^12^	5.13 × 10^13^	*g*_react1_(*α*) = 1 − (1 − *α*)^1/4^
						*g*_react2_(*α*) = (1 − *α*)^−3^ − 1
	VYA/CE	93.82	101.54	6.31 × 10^11^	1.20 × 10^12^	
UPR/OMMT-1	TAS	81.74	87.08	1.26 × 10^9^	1.62 × 10^10^	*g*_react1_(*α*) = 1 − (1 − *α*)^2^
						*g*_react2_ (*α*) = 1 − (1 − *α*)^1/3^
	VYA/CE	81.70	87.05	2.31 × 10^8^	7.08 × 10^10^	
UPR/OMMT-3	TAS	67.53	74.33	6.46 × 10^6^	9.12 × 10^8^	*g*_react1_(*α*) = 1 − (1 − *α*)^2/3^
						*g*_react2_ (*α*) = 1 − (1 − *α*)^1/4^
	VYA/CE	67.46	74.29	4.37 × 10^6^	4.57 × 10^8^	
UPR/OMMT-5	TAS	60.71	72.93	2.75 × 10^5^	5.50 × 10^6^	*g*_react1_(*α*) = 1 − (1 − *α*)^2/3^
						*g*_react2_ (*α*) = (1 − *α*)^−1/2^ − 1
	VYA/CE	60.50	72.88	2.04 × 10^5^	4.17 × 10^6^	

In the redox region, The value of A_1_ in the pristine UPR is approximately 1.41 × 10^12^. However, in UPR/OMMT systems, this value is significantly reduced, ranging from 1.26 × 10^9^ for UPR/OMMT-1 to 2.75 × 10^5^ for UPR/OMMT-5 in the redox region. This reduction can be attributed to the incorporation of nanoclay, increasing the viscosity of the system. The nanoclay platelets absorb styrene molecules, resulting in slower motion of the reactive components.^61^ As a result, the number of collisions between reactive components outside of the nanoclay platelets is reduced.

In the thermal decomposition region of peroxide, due to the gelling process, the viscosity has an insignificant effect on the reduction of collisions. Reduction of styrene concentration in interlayer matrix reduces the styrene linkage between alkyd chains and accordingly the styrene penetration into the gel lattice becomes more difficult, thus the collisions between reactionally components are reduced intensively.^61^ On the other side, the special restriction caused by the incorporation of nanoclay particles led to the reduction of A_2_ in UPR/OMMT systems compared to pristine UPR.

Another important point in the investigation of the curing process of UPR and UPR/OMMT is to analyze the variation of the most probable reaction models *g*(*α*) derived from the isoconversional TAS model. The corresponding mathematical models *g*(*α*) derived for UPR and UPR/OMMT are presented in Table 3. For the first reaction, the obtained *g*(*α*) model indicates that the UPR curing process is properly described by the F_3/4_ chemical process equation. However, after the incorporation of OMMT loads, the chemical process equation is described by F_1/3_ and G1 chemical process equation. For the second reaction, the obtained *g*(*α*) model for the UPR curing process is properly described by F_4_ chemical process. However, after the incorporation of OMMT loads, the chemical process equation is described by F_2/3_, F_1/4_, F_3/4_, and G1 process indicating a shift in the rate-controlling step of curing from a chemical reaction to the diffusion process.

### 3.4. Thermogravimetric analysis results

**Fig. 12:**
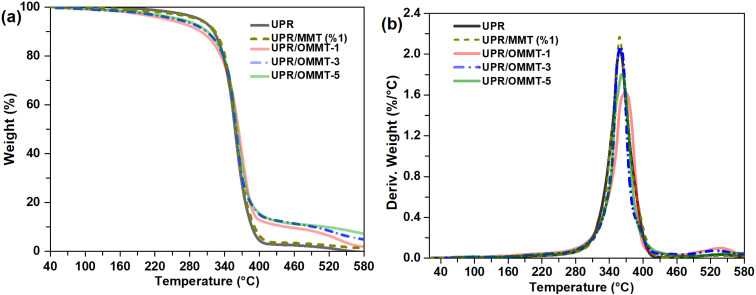
(a) TGA and (b) DTG thermograms of unmodified and modified UPR with MMT and OMMT at different loads.

Further investigation is conducted to examine the impact of incorporating OMMT on the thermal behavior of cured UPR resin using thermogravimetry TGA. The obtained TGA and their corresponding DTG curves are presented in Fig. 12. The onset degradation temperature, which is the temperature at which 10% degradation occurs (*T*_d10_) and is representative of the onset temperature of degradation, as well as the mid-point degradation temperatures (*T*_d50_), are provided in Table 4.

**Table 4:** Thermogravimetric analysis results for unmodified and modified UPR with MMT and OMMT at different loads

Sample	*T*_d10_ (°C)	*T*_d50_ (°C)	*T*_peak_ (°C)	Residual mass (%) at 580 °C
UPR	300.91	358.48	358.17	0.00
UPR/MMT (1%)	298.62	359.67	358.38	1.57
UPR/OMMT-1	247.96	364.53	368.47	1.88
UPR/OMMT-3	265.36	361.23	359.89	4.85
UPR/OMMT-5	271.43	362.54	362.65	7.18

The TGA results show that the pristine UP resin undergoes major decomposition due to the release of free radicals during the scission of the unsaturated polyester backbone.^63^ The UPR/OMMT systems degradation occurs at a faster rate between 200 and 380 °C compared to the unmodified UPR where the onset temperature *T*_d10_ decreased from 300 °C for pristine UPR to 247 °C and 265 °C for UPR/OMMT-1 and UPR/OMMT-3 systems, respectively. This weight loss observed in the mentioned temperature range is likely due to the degradation of the intercalated organic compound as well as water on the clay surface and that between silicate layers.

The thermal degradation of the three UPR/OMMT systems exhibits a delay beyond 380 °C, whereas the UPR/MMT system behaves similarly to the unmodified UPR. The mid-point degradation temperatures experience a marginal rise of approximately 6 °C for the UPR/OMMT systems, and this increase becomes more pronounced at elevated temperatures.

Table 4 displays the maximum temperature values (*T*_peak_) obtained from the first derivative of the weight loss. These values indicate the temperature at which the maximum rate of weight loss occurs. The maximum temperatures of the derivative curve of UPR/MMT remain unchanged. However, they increase for the UPR/OMMT systems to 10 °C in the case of UPR/MMT-1. All the UPR/OMMT systems exhibit a much slower degradation rate and a relatively broad peak at their maximum weight loss temperature compared to neat UPR. This behavior can be attributed to the promotion of polymerization from inside the clay galleries and also from its surface/edges. The reactive double bonds present in the intercalation compound bonded to the clay contribute to polymerization, leading to a decrease in the degradation rate of the polymer around the clay surface.^64,65^

The decrease in degradation rate at the maximum weight loss temperature for UPR/OMMT systems can be explained by two factors. Firstly, the compact silicate matrix in multi-layered intercalated systems causes hindered out-diffusion of the volatile decomposition products or at least a slower departure from interlayer galleries. This results in a reduction in the permeability or diffusivity of volatile degradation products^66,67^ Secondly, the effective dispersion of clay in the unsaturated polyester resin leads to a maximized interaction between the clay and the polymer matrix because of a larger surface area of the clay interacting with the polymer. This interaction leads to restricted molecular mobility of the polymer chains and results in the inhibition of the diffusion of the decomposition products in the polymer matrix.^68^

## 4. Conclusions

During this study, we investigated the efficiency of repetitive organic modification of montmorillonite nanoclay using benzododecinium chloride salts. The study found that the organic modification of montmorillonite had a potential impact on the curing kinetics and thermal degradation of unsaturated polyester resin. The integration of organic modifiers within the montmorillonite galleries was confirmed by structural and morphological analyses. The curing behavior of the unsaturated polyester resin containing organically modified clay was investigated using dynamic differential scanning calorimetry (DSC), followed by kinetic analysis using isoconversional methods. The dynamic DSC curing curves showed a bimodal exothermic peak, indicating two independent reactions, namely, redox and thermal decomposition of the initiator. The incorporation of nanoclay into unsaturated polyester resin (UPR) resulted in a reduction in the activation energy for both the redox and thermal reactions. Additionally, the addition of organically modified montmorillonite caused a significant decrease in the pre-exponential factor. The thermogravimetric analysis showed that UPR/OMMT systems were more stable than pure UPR.

## Conflicts of interest

The authors declare no potential conflicts of interest with respect to the research, authorship, and/or publication of this article.

## Supplementary Material

RA-014-D3RA06076D-s001
