# Supplementary material for: Unravelling the thermal behavior and kinetics of unsaturated polyester resin supplemented with organo-nanoclay
Source: RSC Adv. 2024 Jan 2;14(1):517–28. doi: 10.1039/d3ra06076d (PMC10759040; doi:10.1039/d3ra06076d)

Electronic Supplementary Material (ESI) for RSC Advances.
This journal is © The Royal Society of Chemistry 2023

# Supplementary materials

## Global heat flow and conversion Vs temperature

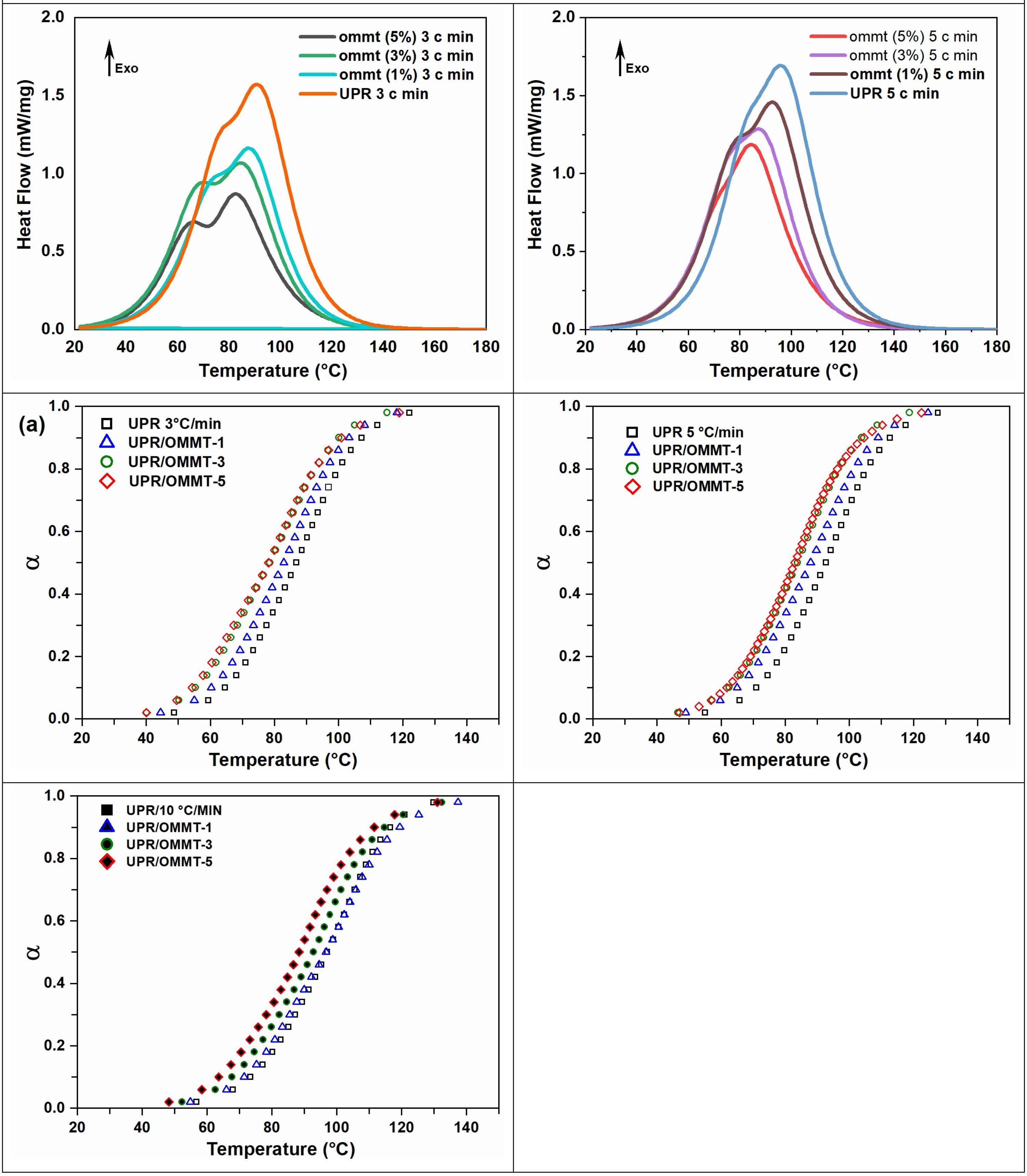

## Ox-red reaction of peroxyde at loaw temperatue

### Log (A1) Vs conversion (TAS)

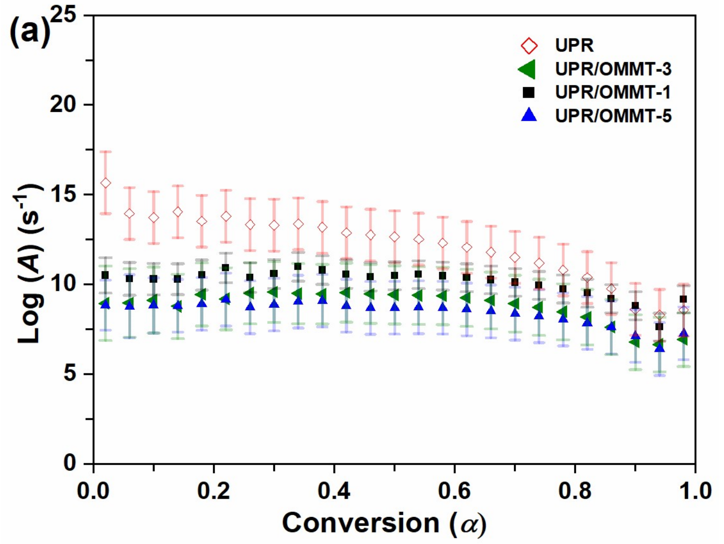


### Log (A1) Vs conversion (VYA/CE 3°C/min)

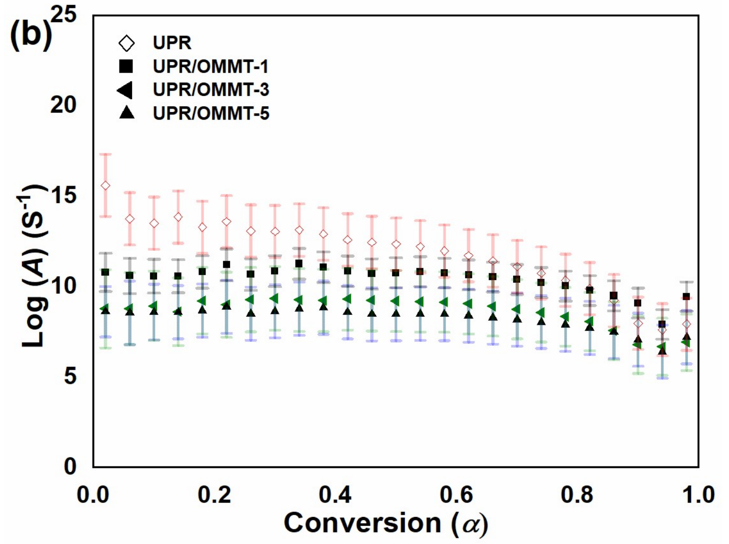


### Log (A1) Vs conversion (VYA/CE 5°C/min)

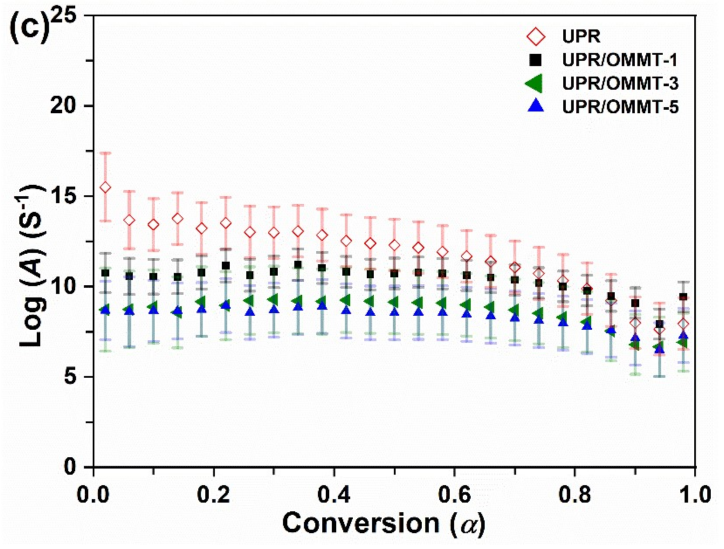


### Log (A1) Vs conversion (VYA/CE 10°C/min)

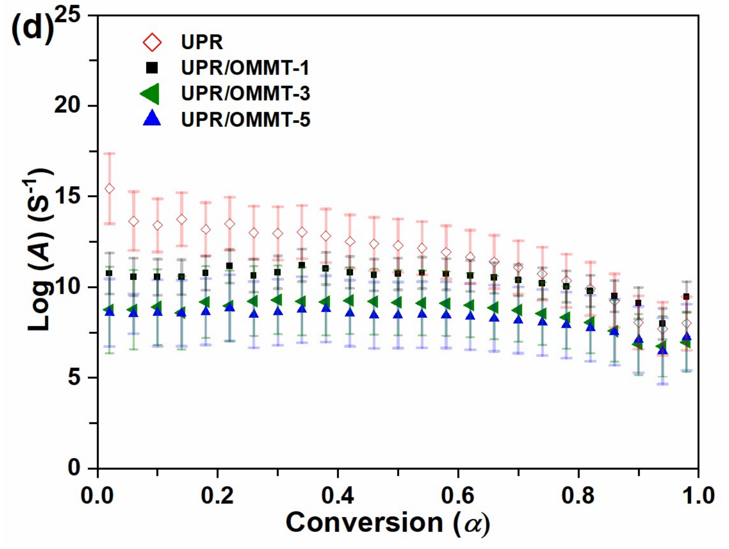

**Thermal decomposition of peroxide at high temperature**

**Log (A2) Vs conversion (TAS)**

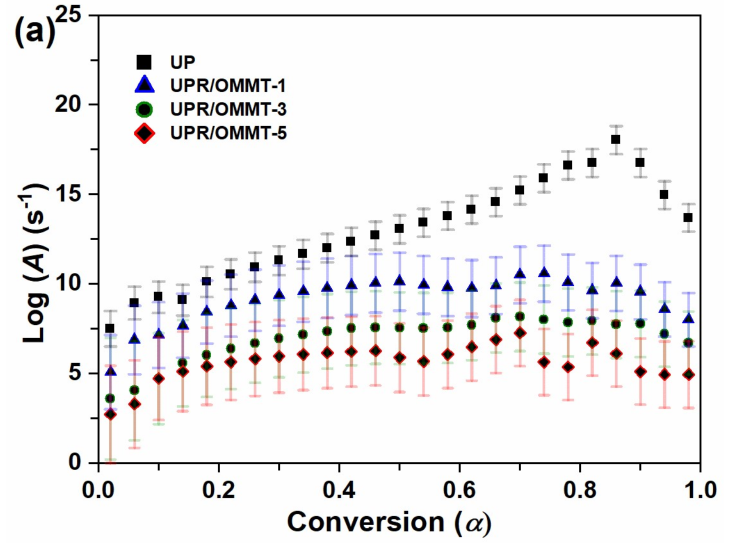


**Log (A2) Vs conversion (VYA/CE 3°C/min)**

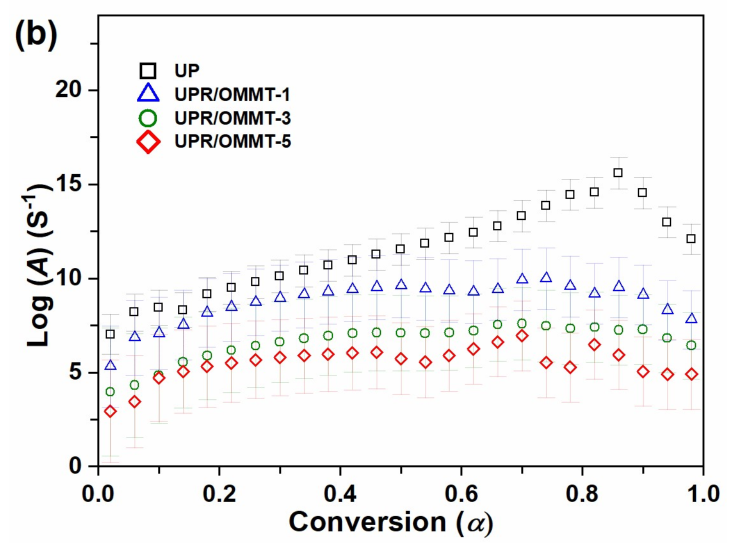


**Log (A2) Vs conversion (VYA/CE 5°C/min)**

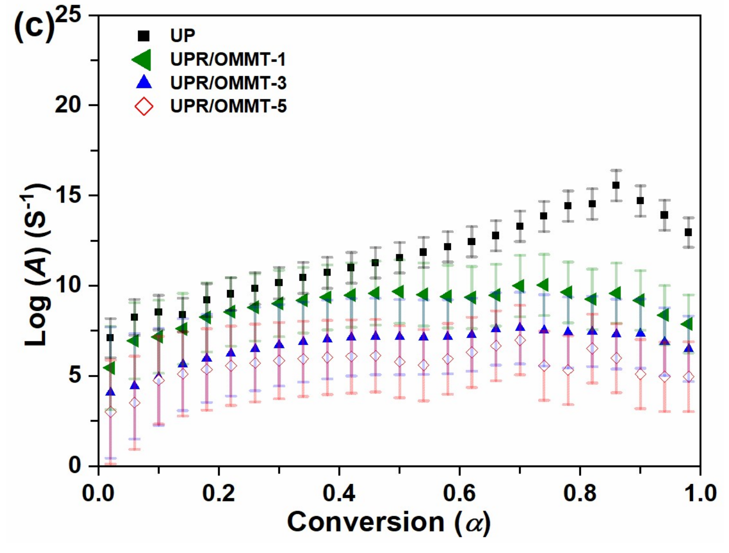


**Log (A2) Vs conversion (VYA/CE 10°C/min)**

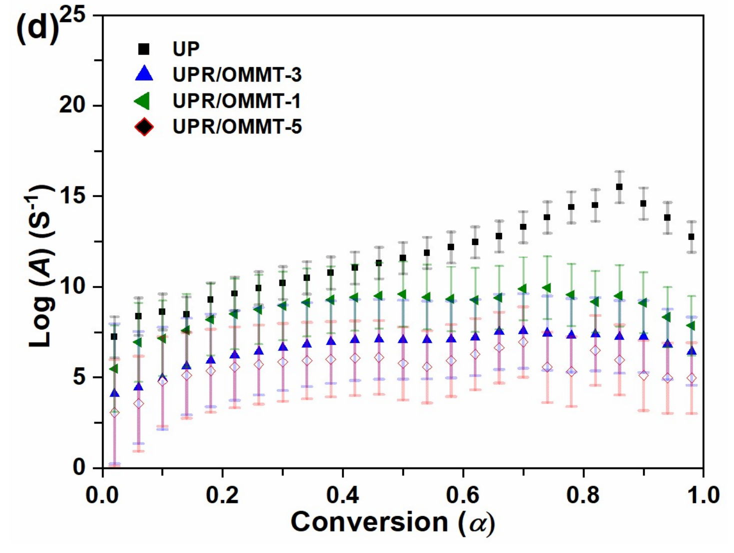

Supplement: RA-014-D3RA06076D-s001 [file RA-014-D3RA06076D-s001.pdf]
